# Morphology and ITS sequences provide insights into the phylogeny of *Tongoloa* (Apiaceae) from China

**DOI:** 10.1186/s12862-024-02292-5

**Published:** 2024-07-30

**Authors:** Lingjian Gui, Chang Peng, Liying Yu, Lijia Liu, Shugen Wei, Zhigang Yan, Xiaomei Zhang, Songdong Zhou, Xingjin He

**Affiliations:** 1 Key Laboratory of Bio-Resources and Eco-Environment of Ministry of Education, College of Life Sciences, Sichuan University, Chengdu, 610065 China; 2 Guangxi Key Laboratory of High-Quality Formation and Utilization of Dao-di Herbs, Guangxi Botanical Garden of Medicinal Plants, Nanning, 530023 China; 3 Guangxi Botanical Garden of Medicinal Plants, Nanning, 530023 China

**Keywords:** *Tongoloa*, ITS, Fruit, Phylogeny, Taxonomy

## Abstract

**Background:**

*Tongoloa* is a genus comprising approximately 20 species, primarily distributed in the mountainous regions of southwest China. The insufficiency of specimen materials and morphological similarities among species render it a taxonomically challenging genus within the Apiaceae family. To elucidate the phylogenetic relationships and taxonomy of Chinese *Tongoloa*, this study utilized a total of 115 nrITS sequences, including 47 recently obtained sequences, for phylogenetic reconstruction.

**Results:**

Phylogenetic relationships reconstructed from ITS sequences indicate that the East Asia Clade and the *Komarovia* Clade are sister groups, and *Tongoloa* belongs to the East Asia Clade. Species of *Tongoloa* are subdivided into 3 distinct groups, all sharing similar fruit morphologies and are clearly differentiated from related taxa. Several *Tongoloa*-like members classified under other genera are interpreted to be closely related to *Tongoloa*. Morphological and molecular data indicate that *Tongoloa*, *Sinolimprichtia* subclade and Chinese *Trachydium* subclade are separate yet genetically contiguous taxa. It is confirmed that *Tongoloa zhongdianensis* belongs to the *Hymenidium* Clade, while *Sinocarum* is classified within the *Acronema* Clade. Two new taxa are found in the Hengduan Mountains.

**Conclusion:**

*Tongoloa* is a genus within the East Asia Clade of Apiaceae, and the phylogeny reconstructed based on ITS sequences divides it into 3 main groups. By integrating fruit morphology and molecular phylogenetic analyses, we preliminary clarified the intricate taxonomic relationships among *Tongoloa* and related taxa. These results provide valuable opportunities for a deeper understanding of the phylogeny of *Tongoloa*.

**Supplementary Information:**

The online version contains supplementary material available at 10.1186/s12862-024-02292-5.

## Background

*Tongoloa* H.Wolff [1] is a genus including about 20 species distributed in the mountainous regions of East Asia [2], especially in southwest China [3, 4]. Most species are confined to alpine meadows, grasslands, shrubs, and forests ranging from 2000 to 4500 m [1, 3, 5–7]. Owing to their distribution in remote mountainous areas with limited accessibility, few of the previously collected specimens have contained mature fruits, which are considered to hold crucial taxonomic value in Apiaceae species [8–12]. Insufficient specimen materials and similar morphologies make this genus taxonomically challenging [13]. Pan and Watson stated that the current taxonomic treatment of *Tongoloa* should be considered tentative [3]. Most Chinese species are still not fully recognized, and the general delimitation between *Meeboldia*, *Sinoodielsia*, *Tongoloa* and *Vicatia* has been problematic and controversial [14].

Over the past 20 years, scholars have endeavored to reconstruct the phylogenetic framework of Apiaceae using molecular methods, aiming to establish a more objective and robust classification system [15–19]. Valiejo-Roman et al. reconstructed the phylogeny of Apioideae (Apiaceae) from the Himalayas by incorporating 13 newly obtained nrITS (nuclear ribosomal internal transcribed spacer) sequences, and the findings indicated that *Tongoloa* is related to *Trachydium simplicifolium* W. W. Smith and *Hansenia* Turcz. [20]. After enriching samples and molecular makers of Apioideae from China, Zhou et al. proposed that *Tongoloa* exhibits a close relationship with specific species of *Sinocarum* H. Wolff ex R. H. Shan & F. T. Pu, *Pimpinella* L., *Trachydium* Lindl. and *Sinolimprichtia* H. Wolff, all residing within the East Asia Clade [21, 22]. Nevertheless, Xiao et al. argued that the genus *Sinocarum* does not belong to the East Asia Clade [23]. *Pimpinella purpurea* (Franch.) H. Boissieu was molecularly closely related to members of *Tongoloa* [24]. It was found that *T. zhongdianensis* S.L. Liou may related to *Hymenidium* Lindl. [25]. Unfortunately, previous molecular analyses often lacked convincing morphological evidence. Examining the vouchers of previous molecular data is rather difficult, as they are usually scattered across herbariums in different regions.

China is the primary distribution area of *Tongoloa*, with all known species and a diverse range of morphological types [3, 26]. Recent field surveys in the Hengduan Mountains have led to a new understanding of species diversity of this genus [27, 28]. However, a comprehensive phylogenetic analysis of *Tongoloa* has yet to be conducted. Owing to its moderate length and rapid mutation rate, the ITS sequence serves as an efficient tool for analysis and validates the accuracy of species morphological taxonomy [17, 21, 29–32]. Additionally, the ITS sequence boasts a remarkable sequencing success rate and offers a cost-effective solution, significantly contributing to the accumulation of extensive species sequences in the current public database. In this study, we adopted extensive sampling and selected the ITS sequence as the molecular marker for phylogenetic reconstruction. Furthermore, we integrated observing and comparative analyses of fruit morphology to determine a more rational phylogenetic relationship for this genus.

## Materials and methods

### Taxon sampling

To examine the morphological variations of *Tongoloa* species, a comprehensive study was conducted on a diverse range of specimens preserved in herbariums A, B, CDBI, E, G, GB, GH, HNWP, K, KUN, LD, MW, NAS, NY, P, PE, SZ, US, W, and WUK. Samples were preferentially collected from or near the type localities of the specimens to ensure accurate and reliable identification. The species identifcation was undertaken by Lingjian Gui and Xingjin He (Sichuan University, Chengdu, China). The nomenclature of taxa was primarily based on Flora of China [3] and the updated checklist of Chinese Apiaceae [26]. Voucher specimens were stored in the herbarium of Sichuan University (SZ) in Chengdu, China (Table 1). The fruits of *Tongoloa* and related taxa were observed and photographed using a Nikon SMZ25 stereomicroscope (Japan).

**Table 1:** Voucher of *Tongoloa* and related taxa collected by us

Species	Voucher	Locality	GenBank number
*Hymenidium lhasanum* Pimenov et Kljuykov	GLJ19092401#	China, Qinghai, Nangqian to Dongba Township	MT124611
*Hymenidium virgatum* Pimenov et Kljuykov	GLJ18082703	China, Sichuan, Xiangcheng to Ranwu Township	MZ054147
	GLJ18091701	China, Sichuan, Xiaojin, Balangshan	MZ054148
*Pimpinella purpurea* (Franch.) H. Boissieu	GLJ18082202#	China, Yunnan, Shangri-La, Xiajisha	MT124604
*Pleurospermum amabile* Craib et W. W. Smith	GLJ19100605#	China, Yunnan, Deqin, Baima Snow Mountain	MT124614
*Sinocarum muliensis* L.J. Gui, Y.P. Xiao & X.J. He	GLJ18081101	China, Sichuan, Muli, Kangwumuchang	MZ054145
*Sinolimprichtia alpina* H. Wolff	GLJ19100702#	China, Yunnan, Deqin, Baima Snow Mountain	MT124613
	LH2018081402	China, Sichuan, Yajiang, Jianziwanshan	MT124609
*Tognoloa tagongensis* L.J. Gui et X.J. He	GLJ18092101	China, Sichuan, Kangding, Tagong	MN630613
	GLJ18102401#	China, Sichuan, Kangding, Tagong	OP422508
*Tongoloa arguta* L.J.Gui & X.J.He	GLJ18082102	China, Yunnan, Deqin, Baima Snow Mountain Pass	MT124599
	GLJ18092002	China, Sichuan, Yajiang, Kazilashan	MT124615
	GLJ19092802#	China, Sichuan, Yajiang, Jiangziwanshan Pass, the old road	MT124612
	xyp A11	China, Yunnan, Shangri-La, Daxueshan Pass	MT124619
	B18	China, Sichuan, Kangding, Zheduoshan Pass	MZ054154
*Tongoloa* sp.	GLJ20071601	China, Sichuan, Xinlong, 13 to 16 km west of Tongxiao	MZ054152
	GLJ20102302#	China, Sichuan, Xinlong, about 13 km west of Tongxiao	OP422516
*Tongoloa dunnii* (H. Boissieu) H. Wolff	GLJ18091102#	China, Hubei, Shennongjia	MT124601
*Tongoloa elata* H. Wolff	GLJ19080404	China, Sichuan, Songpan, Huangshengguan	MT124607
*Tongoloa filicaudicis* Fu Kuntsun	GLJ18102003#	China, Gansu, Wenxian County, Songpingmuchang	MT124600
*Tongoloa fortunatii* (H. Boissieu) Pimenov et Kljuykov	GLJ18090301	China, Fujian, Taining, Huangyanfeng	OP422509
	GLJ18090802-2	China, Jiangxi, Lushan mountain	MN630614
*Tongoloa gracilis* H. Wolff	GLJ18082104	China, Yunnan, Deqin, Baima Snow Mountain Pass	MT124602
	GLJ18082604	China, Sichuan, Xiangcheng, Wumingshan to Maxionggou	OP422507
	J18KD	China, Sichuan, Kangding	MN630616
	LH2018082406	China, Sichuan, Dege, Gengqing	MN630617
	GLJ20071701	China, Sichuan, Xinlong, Jialaxigou	MZ054153
*Tongoloa loloensis* (Franch.) H. Wolff	GLJ18103002-1#	China, Yunnan, Dali, Eryuan, Baicaoluo	MN630615
	LCK20211030003	China, Yunnan, Dali, Eryuan, Luopingshan	OP422510
*Tongoloa napifera* (H. Wolff) C. Norman	GLJ19080401	China, Sichuan, Songpan, Xueshanliangzi	MT124605
*Tongoloa rubronervis* Liou Shoulu	GLJ18080801-2	China, Sichuan, Muli, Kangwuliangzi	MN630618
	GLJ18081002	China, Sichuan, Muli, Guangtoushan	MN630619
*Tongoloa silaifolia* (H. Boissieu) H. Wolff	JQP19081607-2	China, Chongqing, Chengkou	MT124617
	LH2018072802	China, Shaanxi, Meixian, Taibai Mountain	MT124616
*Tongoloa spathulata* sp. nov.	GLJ19092201-1#	China, Qinghai, Yushu, Jiangxigou, Jiangxi Forest Farm	MT124610
	GLJ19092201-2	China, Qinghai, Yushu, Jiangxigou, Jiangxi Forest Farm	OP422513
*Tongoloa taeniophylla* (H. Boissieu) H. Wolff	GLJ18082902#	China, Sichuan, Kangding, Paomashan	MT124598
*Tongoloa xinlongensis* sp. nov.	GLJ20071101	China, Sichuan, Xinlong, Xionglongxi Township	MZ054149
	GLJ20071201	China, Sichuan, Xinlong, Picha Township, Guoba Village	MZ054151
	GLJ20071901	China, Sichuan, Xinlong, Xionglongxi Township	MZ054150
*Tongoloa zhongdianensis* S. L. Liou	GLJ19100501#	China, Yunnan, Shangri-La, Mianshaba	MT124620
*Trachydium simplicifolium* W. W. Smith	GLJ19111401#	China, Yunnan, Lijiang, Yulong Snow Mountain, Mahuangba	MT124618
*Trachydium souliei* H. Boissieu	GLJ19100704	China, Yunnan, Deqin, Baima Snow Mountain	OP422515
	GLJ18082103	China, Yunnan, Deqin, Baima Snow Mountain Pass	MT124603
	GLJ19100604#	China, Yunnan, Deqin, Baima Snow Mountain Pass	OP422511
*Trachydium variabile* H. Wolff	GLJ19080402	China, Sichuan, Songpan, Xueshanliangzi	MT124606
	GLJ19080601	China, Sichuan, Hongyuan, Longriba	MT124608

# specimen with fruit materials

### DNA extraction and sequencing

Total DNA was extracted from silica-dried leaves using the plant genomic DNA kit (CWBIO, Beijing, China). The extracted total DNA was stored in a refrigerator at -20°C. The primers ITS4: 5’-TCC TCC GCT TAT TGA TAT GC-3’ and ITS5: 5’-GGA AGT AAA AGT CGT AAC AAG G-3’, designed by White et al., were selected for PCR amplification of the target sequence [33]. PCR was performed in a 30 µl amplification system, comprising 2 µL total DNA, 9 µl water, 1.5 µL forward primer, 1.5 µL reverse primer and 15 µl Taq PCR Master mix (CWBIO, Beijing, China). All PCR amplifications were performed on a Geneamp PCR System 9700 (USA). The ITS amplification protocol involved an initial denaturation step for 2 min at 95 °C, followed by 35 cycles of 60 s at 94 °C, 45 s at 52.5 °C, and 60 s at 72 °C, and a final extension step of 7 min at 72 °C. Bidirectional sequences were assembled using SeqMan in DNASTAR 5.01 [34], with subsequent necessary manual inspection. The completed ITS sequences and the boundaries of ITS1 and ITS2 were determined by comparison with the ITS sequences of *Tongoloa* available on NCBI.

### Sequence alignment and phylogenetic analysis

A total of 115 ITS sequences of Apiaceae were used for constructing the phylogenetic tree. 47 ITS sequences are recently obtained and the voucher information is presented in Table 1, while the remaining 68 sequences are downloaded from NCBI (Table S1). The ITS dataset includes 48 species from the East Asia Clade, 7 species from the *Komarovia* Clade, 14 species from the *Acronema* Clade, 4 species from the *Hymenidium* Clade (*Sinodielsia* Clade), and 5 species from Pleurospermeae [17]. All sequences were aligned using MAFFT [35]. The aligned sequences were manually inspected and the phylogenetic analysis was conducted using maximum likelihood (ML) and bayesian inference (BI) methods. The optimal DNA model was selected in Mega7 [36], and a ML tree with 500 bootstraps was subsequently constructed. Mrbayes v3.2 [37] was employed to infer a BI tree with 5 million generations using the GTR + G model. The first 25% of the trees were discarded, and the remaining trees were utilized to calculate posterior probability (PP) statistics. Bootstrap (BS) and PP values were annotated on the branches of a ML tree. Jalview2 [38] was used to visualize sequence base variations.

## Results

### Fruit characteristics

**Fig. 1:**
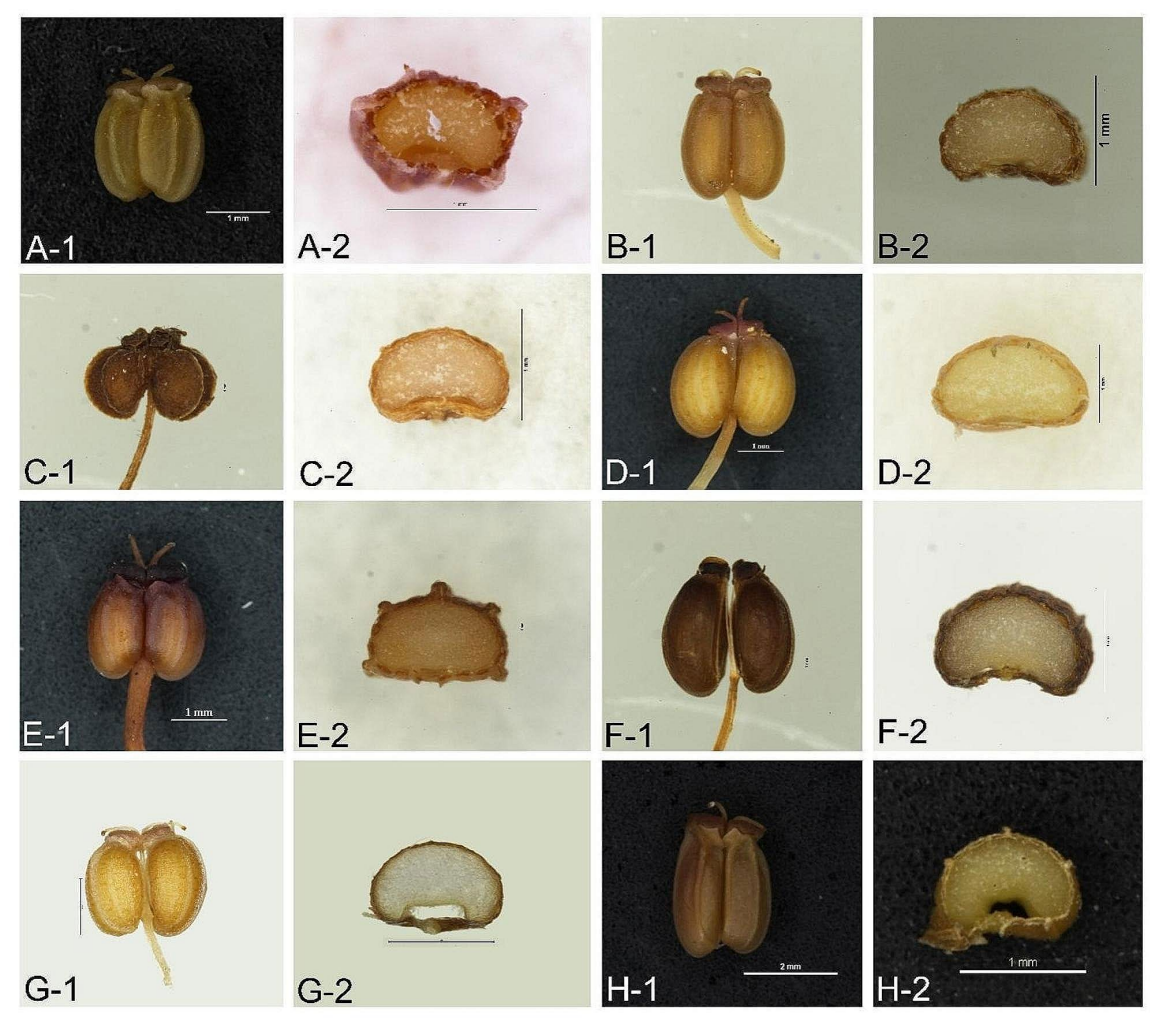
Fruit morphology of *Tongoloa* species. (**A**) *T. tagongensis* (GLJ18092101 & GLJ18102401); (**B**) *T. elata* (GLJ18101802); (**C**) *T. filicaudicis* (GLJ18102003); (**D**) *T. loloensis* (GLJ18103002); (**E**) *T. arguta* (GLJ19100602); (**F**) *T. silaifolia* (rt20092320); (**G**) *T. taeniophylla* (GLJ18082902); (**H**) *T. gracilis* (GLJ18092102). (1) Lateral view of the cremocarp; (2) Cross-section view of the mericarp. The items within the parentheses are voucher specimens

The fruit morphology and anatomical features of *Tongoloa* were studied. We found that these fruits are primarily wide oval with a cordate base, exhibiting filiform ribs, 3 vittae in each furrow, and 4 vittae on the commissure. The stylopodium is depressed, while the ventral surface of the endosperm is flat-concave (Fig. 1). We subsequently examined the fruit morphologies of genera that have recently been considered closely related to *Tongoloa* in molecular phylogeny [21, 22]. The results revealed that the fruits of *Pimpinella purpurea* (Fig. S2) and *Trachydium variabile* (Fig. S3) exhibit a cordate base, 5 filiform ribs, 3 vittae in each furrow, and 4 vittae on the commissure. Additionally, they possess a depressed stylopodium, and a ventrally flat-concave endosperm, featuring closely align with those observed in common *Tongoloa* species. The morphological results support the conclusion that *Pimpinella purpurea* and *Trachydium variabile* may be closely related to *Tongoloa* [22]. However, in contrast, the fruits of *Sinolimprichtia alpina* exhibit a long obovate shape with a narrowed base and a fluffy peel, and possess 6 vittae on the commissures (Fig. S1 A). These features are significantly different from those of *Tongoloa*. Similarly, the fruits of *Trachydium souliei* are obovate with a narrowed base and wing-like ribs, and also display 6 vittae on the commissures (Fig. S1 D), distinguishing them from *Tongoloa* fruits. The mature fruits of *T. zhongdianensis* are distinctly dissimilar to those of the genus *Tongoloa* (Fig. S4).

### Phylogenetic analysis

**Fig. 2:**
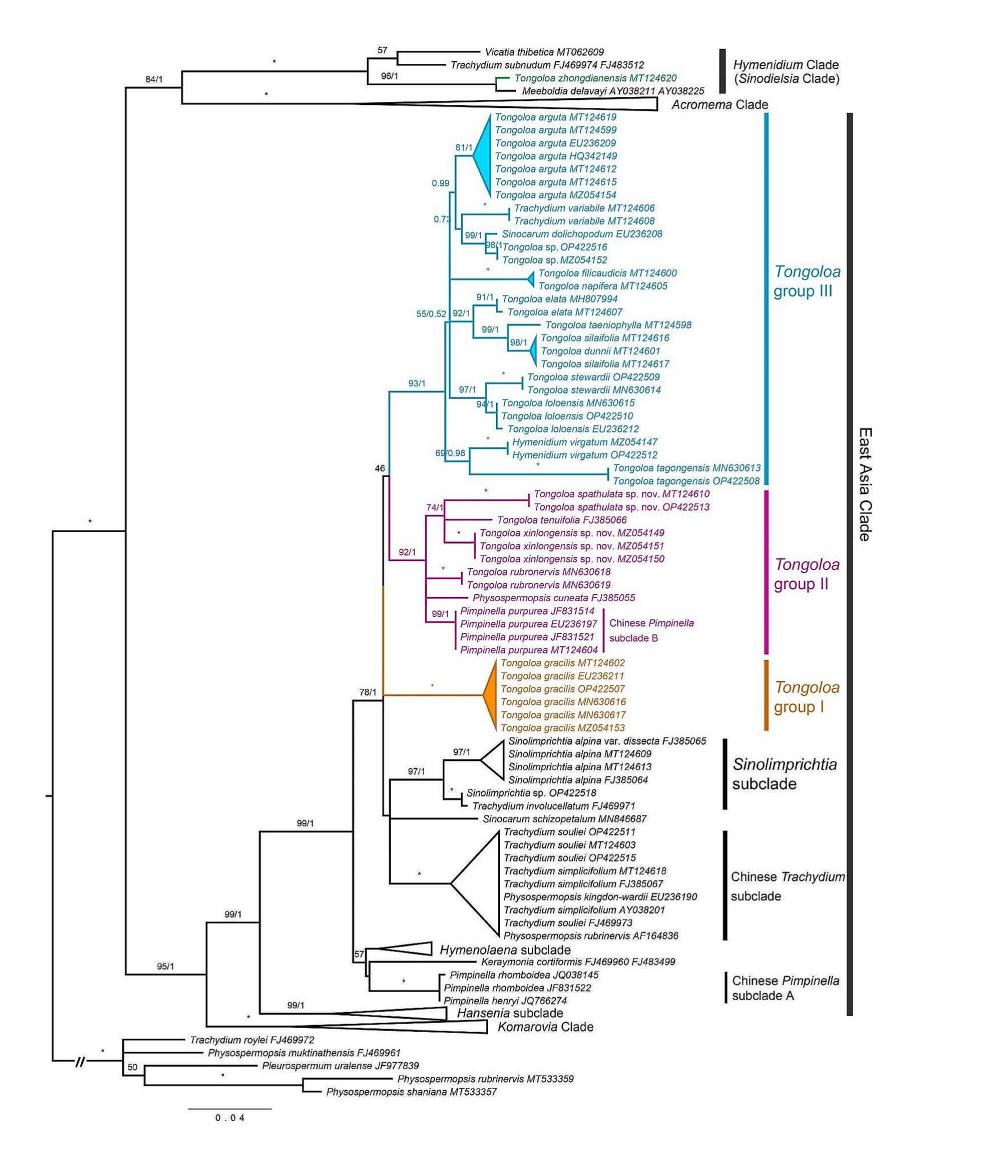
Phylogram obtained through maximum likelihood analysis based on ITS sequences. *Tongoloa* is positioned within East Asia Clade and is divided into 3 groups. The BS/PP values are indicated on the branches. When both values are at their maximum, they are marked with an asterisk (*). Values below 50% and 0.5 are typically omitted

The ML consensus tree and BI tree have similar topological structures. The East Asia Clade and the *Komarovia* Clade are sister groups with strong support. Phylogenetic analysis using both ML and BI methods divides *Tongoloa* into three clustered groups (Fig. 2), although some branches require additional reinforcement for stronger phylogenetic support. Group I includes only one species, *T. gracilis*. Group II comprises at least 6 species, including *Pimpinella purpurea*, *Physospermopsis cuneata* H. Wolff, *Tongoloa rubronervis*, and 2 potential new species (*T. spathulata* nov. sp. and *T. xinlongensis* nov. sp.). Group III has at least 10 species, including *T. tagongensis*, *T. loloensis*, *T. fortunatii*, *T. silaifolia*, *T. taeniophylla*, *T. elata*, *T. napifera*, *T. arguta*, *Trachydium variabile* and *Hymenidium virgatum*. Notably, while the *Tongoloa* groups, the *Sinolimprichtia* subclade and the Chinese *Trachydium* subclade are each recognized as separate taxa on the phylogenetic tree, their branching patterns reveal remarkably close affinities, suggesting complex speciation or recent divergence within the broader evolutionary context. *Tongoloa zhongdianensis* is not located within the East Asia Clade, but it exhibits a significant association with taxa belonging to the *Hymenidium* Clade.

### Sequence comparative analysis

**Fig. 3:**
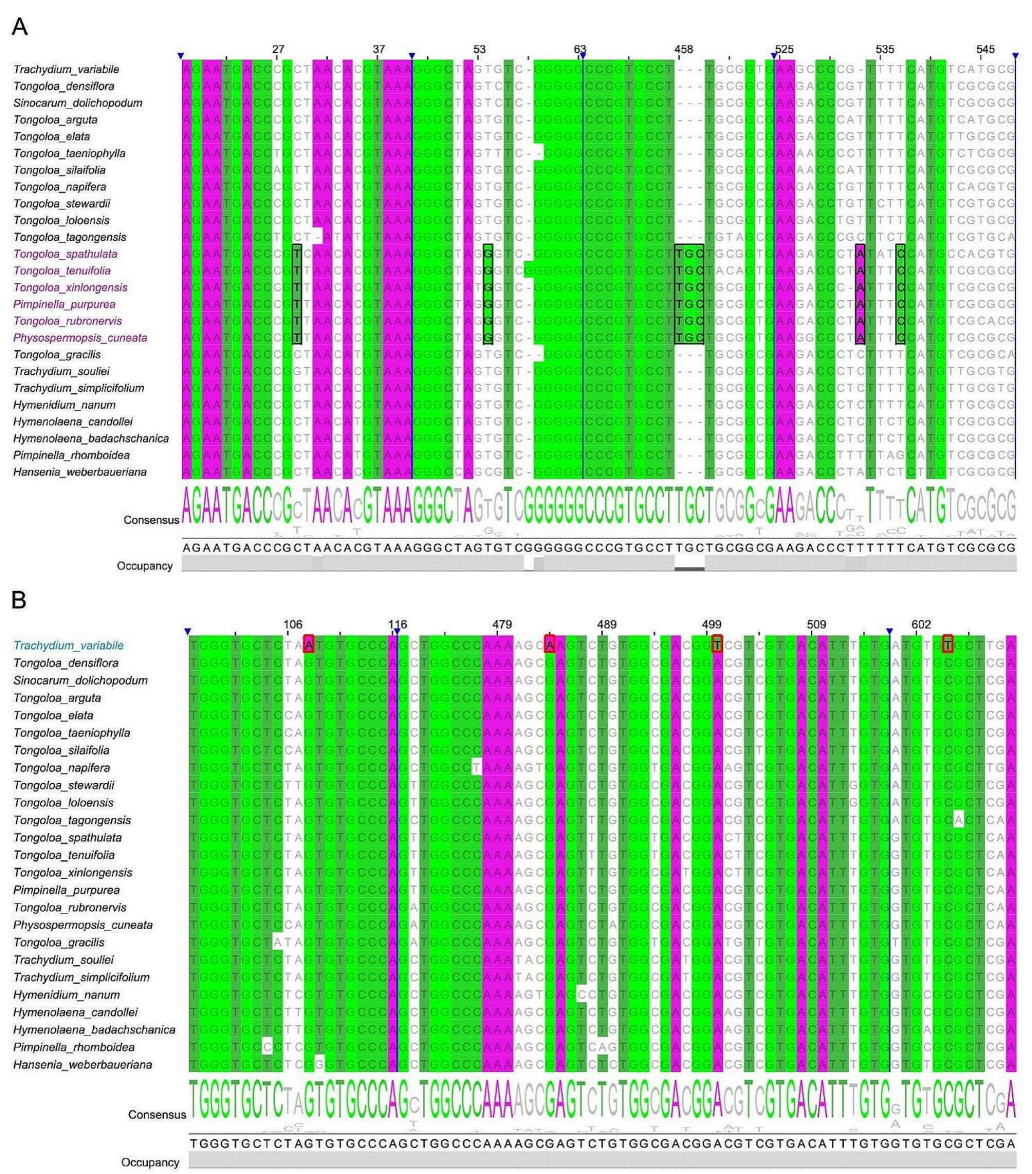
Comparison of aligned ITS sequences of 24 taxa from East Asia Clade. (**A**) *Pimpinella purpurea* shares common variations at loci 457–460 bp, 533 bp, 537 bp and 610 bp with other species in *Tongoloa* group II (indicated by purple font and black boxes); (**B**) The specific variations of *Trachydium variabile*, relative to closely related species, are found at loci 108 bp, 484 bp, 500 bp and 605 bp (indicated by blue font and red boxes)

A comparative analysis was performed on the ITS sequences of 13 *Tongoloa* species and 14 other closely related species from the East Asia Clade. The ITS sequence of *Pimpinella purpurea* exhibits identical specific base variations at positions 457–460 bp, 533 bp, 537 bp, and 610 bp with 4 closest *Tongoloa* or *Tongoloa*-like species (Fig. 3A). The ITS sequence of *Trachydium variabile* shows a strong similarity to that of closely related *Tongoloa* species, yet it displays notable variations at loci 108 bp, 484 bp, 500 bp and 605 bp (Fig. 3B).

## Discussion

### Phylogeny and taxonomy of *Tongoloa*

As of now, ITS sequences remain the principal criterion for defining the major clades and tribes of the Apiaceae family [17]. Furthermore, the processing of precursor rRNA transcripts is a remarkably conserved biological mechanism. The secondary structure of the ITS in nuclear rRNA transcripts is widely documented across eukaryotes, and this feature has been employed to facilitate phylogenetic comparisons [39].

Based on the most extensive molecular sampling of *Tongoloa* and its closest relatives to date, it has been confirmed that *Tongoloa* belongs to the East Asia Clade of Apiaceae [17]. The internal taxa of *Tongoloa* have been tentatively divided into three groups, including common *Tongoloa* species and some *Tongoloa*-like species, with the exception of *T. zhongdianensis*. *Pimpinella purpurea* and *Physospermopsis cuneata* are classified under *Tongoloa* group II, while *Trachydium variabile* falls under *Tongoloa* group III (Fig. 2). Morphological studies reveal that most of the species within the three groups, including those temporarily listed in other genera, share similar characteristics. These features include conical roots, slender stems, 3-ternate/pinnate leaves, depressed stylopodium, flat petals, fruits with a broadly ovoid shape and cordate bases, 5 filiform ribs, 3 vittae in each furrow and 4 vittae on the commissure. Additionally, the seed faces exhibit a flat-concave appearance. These shared characteristics support the classification of *Tongoloa* as an independent genus.

Previous studies have revealed that the phylogenetic relationship between *Tongoloa*, *Sinolimprichtia alpina*, *Trachydium simplicifolium*, and *Trachydium souliei* remains uncertain [21, 22]. In the phylogenetic tree of this study, although the *Tongoloa* groups, the *Sinolimprichtia* subclade, and the Chinese *Trachydium* subclade are almost independent of each other, they are located within the same major clade (Fig. 2), indicating a close genetic relationship. We have newly added fruit morphology data and found that the fruits of the *Sinolimprichtia* subclade and Chinese *Trachydium* subclade usually possess distinctive wing-like ribs and a higher number of vittae on the commissure (Fig. S1), which are noticeably distinct from the features of *Tongoloa*. In recent studies on chloroplast genomes and ribosomal DNA, *Sinolimprichtia alpina* was identified as a likely ancient hybrid taxon [40]. The potential hybridization may introduce ambiguity to the boundaries between *Tongoloa* and its closely related taxa. Furthermore, the ubiquity radiation of plants in the Hengduan Mountains presents challenges to the the division of many generic boundaries [41, 42]. In light of molecular phylogenetic analyses and distinctive fruit morphological traits, acknowledging *Tongoloa* as a separate genus appears to be a justifiable classification strategy. This approach not only reflects the unique evolutionary lineage of *Tongoloa* but also respects its discernible morphological identity, thereby contributing to a more accurate and refined understanding of its taxonomic placement within the family.

Despite our diligent efforts in this study, we are unable to obtain molecular sequences for three dubious *Tongoloa* taxa. The species *T. pauciradiata* H. Wolff, identifiable only through its type specimen collected in Lhasa, Tibet (barcode K000075377), exhibits fruit and plant morphologies similar to advanced Apioideae taxa, diverging from the primitive characteristics of basal lineages such as the East Asia Clade. *Tongoloa rockii* H. Wolff is represented solely by type specimens (barcodes E000000521 and GH00077982) from Yulong Snow Mountain in Yunnan. This species, characterized by its short and slender plants, thick root and 4-pinnate/pinnatifid leaves, is acknowledged as a good species by Fading Pu [43]. After analyzing the morphological features and ITS sequences of similar species, it is evident that its phylogenetic position belongs to the *Tongoloa* group III. The species *T. smithii* H. Wolff (barcode GB-0048829) is exclusively found in its type locality, Maerkang, Sichuan. The distribution of this species lies within 200 km of *T. elata*’s type locality in Huangshengguan, Songpan, Sichuan. Despite lacking fruits on its type specimen, *T. smithii* exhibits morphological similarities in its flowers, leaves, and overall plant structure to *T. elata*, indicating a possible close relationship or even species identity.

Zhou et al. provisionally referred to the *Pimpinella* species in the East Asia Clade as the Chinese *Pimpinella* subclade [21, 22]. However, the findings of this study have underscored the imperative need to further categorize these *Pimpinella* taxa into two separate subclades, temporarily designated as Chinese *Pimpinella* subclade A and Chinese *Pimpinella* subclade B (Fig. 2). Subclade A is phylogenetically distant from *Tongoloa* and exhibits a close relationship with *Keraymonia*. Currently known species include *Pimpinella henryi* Diels and *P. rhomboidea* Diels. The fruit bases of this subclade are cordate, and have 5 filiform ribs, exhibiting some similarity to *Tongoloa*. However, the stylopodium shape, style length, and the number of vittae in the fruit significantly differ from *Tongoloa*. Chinese *Pimpinella* subclade B stands as a pivotal groups in this study. Currently, the confirmed species primarily include *P. purpurea*, whose ITS sequences exhibit a close clustering with species of *Tongoloa* group II. Moreover, morphologically, *Pimpinella purpurea* exhibits an ovoid fruit shape with a cordate base, filiform ribs, 4 commissural vittae, and a flattened stylopodium (Fig. S2), all of which align closely with the morphological features of *Tongoloa*.

*Trachydium variabile* is presently a species lacking extensive morphological and molecular studies, and is not included in Flora of China [43]. We verified the original literature, the type specimens, as well as fresh samples collected from the type locality. The newly collected samples exhibited a remarkable consistency with the type specimens. Additionally, we enhanced the population sample by incorporating specimens from the vicinity of Longriba, Hongyuan, Sichuan. The sequencing results reveal a congruency in the ITS sequences of both populations. The phylogenetic tree constructed on the basis of ITS sequences positioned *T. variabile* within *Tongoloa* group III. In terms of morphology, the flower, fruit, and leaf characteristics of this species are similar to those of the typical species of *Tongoloa* group III (Fig. S3). Analysis revealed that the ITS of *Trachydium variabile* had undergone significant genetic variation compared with related taxa, particularly with four unique base variations at the 108 bp, 484 bp, 500 bp, and 605 bp loci (Fig. 3B). These results hint that *Trachydium variabile* is an independent species in *Tongoloa* Group III.

Also noteworthy, the results of molecular analysis support the suggestion made by Pan and Watson in Flora of China, to classify *Physospermopsis cuneata* as belonging to *Tongoloa*. However, it is unfortunate that we failed to collect samples with mature fruits from the type locality of *Physospermopsis cuneata* in Yulong Snow Mountain, Yunnan. Therefore, it is crucial to supplement the collection materials for future research purposes.

The mature fruits of *Tongoloa zhongdianensis* collected from the type locality exhibit significant morphological differences from *Tongoloa* species (Fig. S4). ITS molecular markers indicate that the systematic position of this species falls within the *Hymenidium* Clade (*Sinodielsia* Clade), possibly having a closer relationship with some species of *Meeboldia*, while being distant from the East Asia Clade where *Tongoloa* resides. Currently, this taxon is still considered an independent species, but its precise taxonomic treatment requires further determination.

*Sinocarum* was previously considered to be closely related to *Tongoloa* [14]. Valiejo-Roman et al. conducted a sampling and phylogenetic study of Apiaceae from the Himalayan region, involving one representative taxon of *Sinocarum* and another representative taxon of *Tongoloa* [20]. The result indicates that despite the morphological similarities between *Sinocarum* and *Tongoloa*, they do not share a close genetic relationship. Recent research, bolstered by our findings, confirms that the majority of *Sinocarum* species, particularly *S. coloratum*, *S. cruciatum* (Franch.) H. Wolff, and *S. vaginatum* H. Wolff, all belonging to the *Acronema* Clade, exhibit significant genetic divergence from *Tongoloa* [23, 44]. The morphological similarity between these two genera may be attributed to convergent evolution resulting from selective pressures in high-altitude environments rather than genetic relationships. Indeed, there are significant morphological differences between the mature fruits of *Sinocarum* [23] and *Tongoloa*. Unfortunately, most of the previously gathered specimens of *Sinocarum*, including type specimens, were devoid of mature fruits, potentially leading to erroneous identifications in the past.

### Two new species of *Tongoloa*

The morphological variation of *Tongoloa* species distributed in the Hengduan Mountains is complex, and specimens are easily misidentified when morphological characteristics are not sufficient [23, 27, 45]. We found a questionable specimen [T. N. Ho (HNWP), B. Bartholomew (CAS), M. Watson (E) and M. Gilbert (MO at BM) 2685] preserved in the Qinghai-Tibetan Plateau Museum of Biology (Xining, Qinghai, China). It was identified as *Tongoloa gracilis*. Nevertheless, the petal tips of this particular plant are blunt instead of pointed, which is not aligned with the typical features of *T. gracilis* with incurved petal tips. In 2019, guided by sampling records, we conducted a field investigation to Jiangxi Forest Farm in Jiangxigou, Yushu, Qinghai Province, and collected the fresh materials of this unusual species on the grassy slope under *Picea* trees. The ITS phylogenetic analysis reveals that this species occupies a distinct branch within *Tongoloa* group II (Fig. 2). We conducted a thorough examination of all available specimens and literature, with a particular emphasis on the taxa from the Qinghai-Tibet Plateau. After a thorough analysis encompassing morphology, nrITS sequence, and geographic distribution, we have determined that this plant belongs to an undescribed species. Given the distinctive morphological characteristics of its petals, we have designated it as *T. spathulata*.

Xinlong County, situated in the Hengduan Mountains of Sichuan Province, boasts an altitude ranging from about 2,700 m to 5,900 m. The continuous mountains here are separated by several river valleys. There have been scant records of Apiaceae specimens in Xinlong County and its neighboring regions (at least until recently). In 2020, during our survey of plant resource in this County, we discovered an special species of *Tongoloa*. The plants preferentially thrive in humid places at altitudes of 3200–3800 m, growing among shrubs or in herb communities. Combined with the geographical distribution, we examined the morphology and molecular marker (ITS) of the newly collected samples (Figs. 2 and 3), and confirmed that this species is closely related to *T. spathulata*, which is distributed in Qinghai. However, the petals, fruits and ITS sequences of this species are significantly different from those of similar taxa, and it cannot be placed in any known *Tongoloa* or other Apiaceae species. Here we formally describe it as a new species, *T. xinlongensis*.

### Taxonomic treatment

#### *Tongoloa spathulata* L.J. Gui & X.J. He, sp. nov

**Diagnosis.** The new species is morphologically similar to *T. taeniophylla* (H. Boissieu) H. Wolff, but distinguished by having spatulate petals (vs. narrow linear petals).

**Type. CHINA**,** Qinghai**: Jiangxi Forest Farm, Jiangxigou, Yushu. East of Jiangxi Forest Farm Station, 3640 m, 32°4′ N, 97°4′ E, 22 Sept. 2019, Ling-jian Gui & Chang Peng, GLJ19092201 (Holotype: SZ!; isotype: PE!); Jiangxigou, Yushu. E of Jiangxi Forest Station, on E side of the Zi Qu and SE of Mozhong. 3640 m, 32°5′ N, 97°4′ E, 29 Aug 1996, T.N. Ho et al. 2685 (Paratype: HNWP!, PE!).

**Fig. 4:**
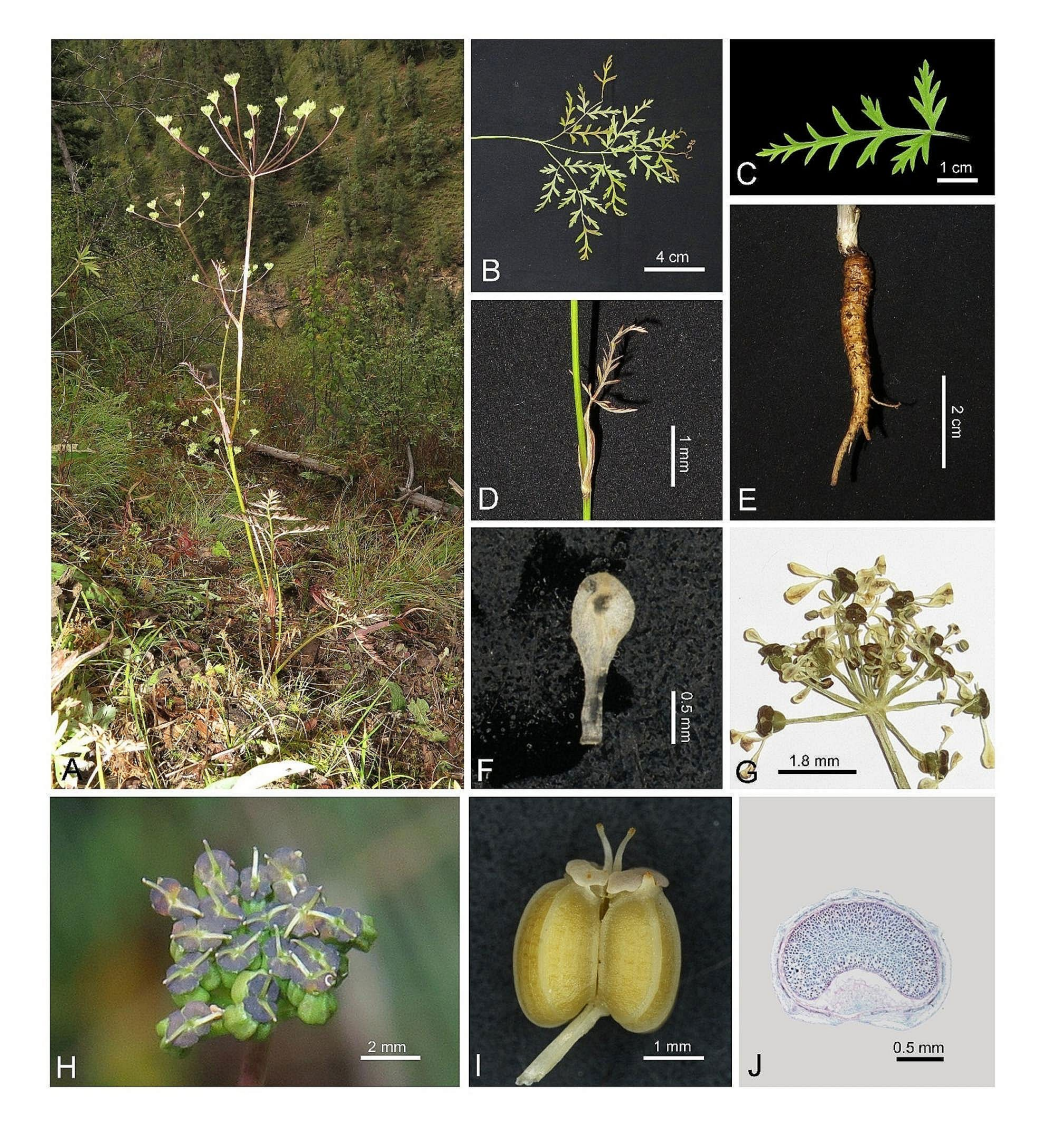
*Tongoloa spathulata* sp. nov. (**A**) Plant and habitat; (**B**) Basal leaf; (**C**) Ultimate segments of basal leaf; (**D**) Upper leaf with sheath-like petiole; (**E**) Root; (**F**) Petal; (**G**) Umbellule; (**H**) Fruit, top view; (**I**) Fruit, Lateral view; (**J**) Mericarp, cross section view

**Fig. 5:**
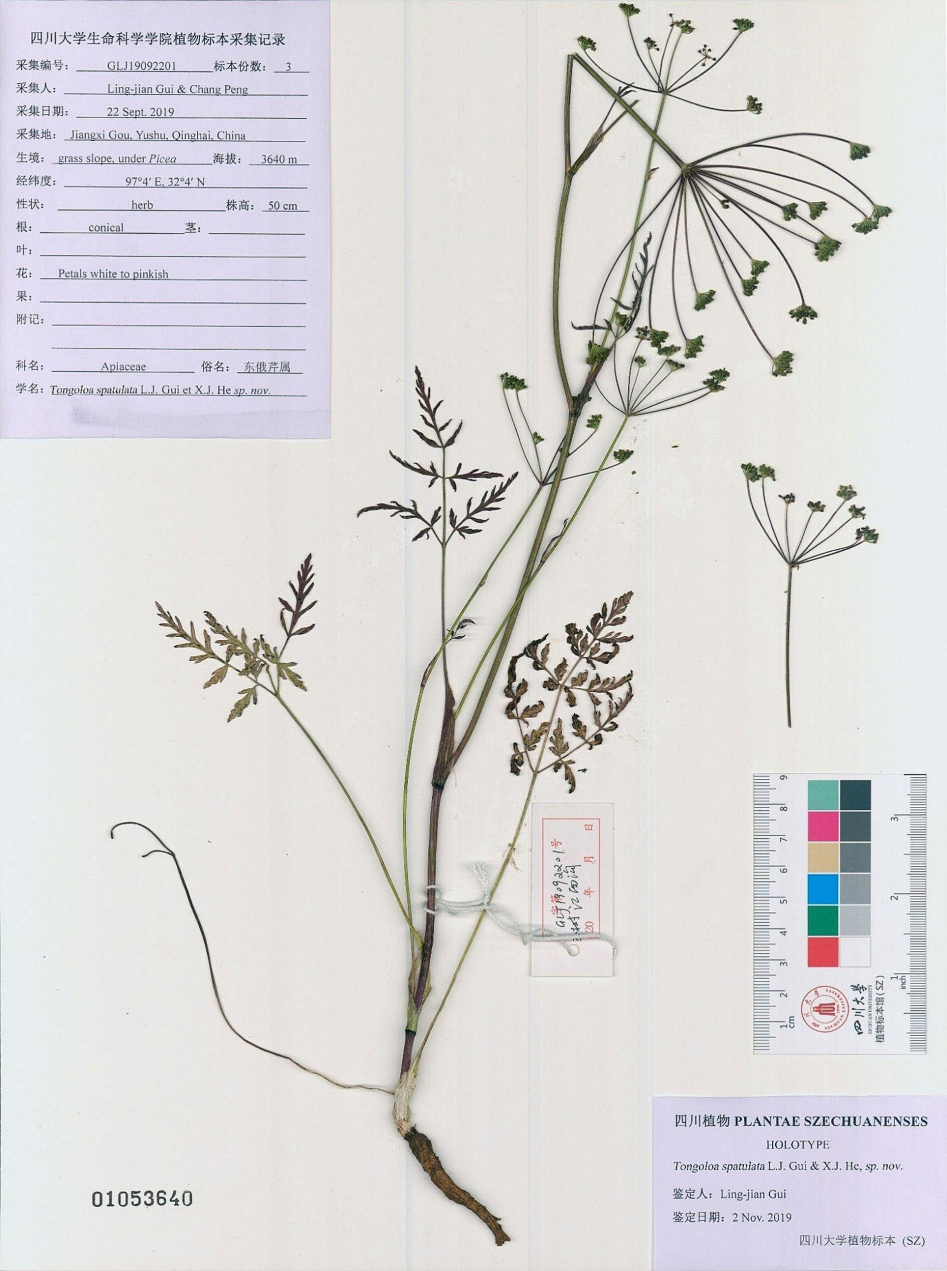
Holotype specimen of *Tongoloa spathulata*

**Description.** Plants 25–80 cm. Root conic, 2–6 × 0.4–1 cm. Stem striate, green to purplish, glabrous, branched. Lower leaves long-petiolate, petioles sheaths inflated, membranous; upper leaves sessile, sheath membranous. Blades triangular, 3-ternate/pinnate; ultimate segments lanceolate, 2–6 × 0.8–2 mm; Terminal peduncle 4–15 cm, lateral peduncle 2–9 cm. Umbels 4–12 cm across; bracts absent; bracteoles absent or 1–6; rays 7–16, 2–7 cm, unequal; umbellules 8–15-flowered, 2–4 mm; Calyx teeth minute, acute; Petals spatulate, white to pinkish, 1–1.5 × 0.4–0.8 mm, apex obtuse-rounded; stylopodium pressed, dark purple; styles short, reflexed outward. Fruit broad–ovoid, ca. 2.2 × 1.8 mm, base cordate; ribs 5, filiform to inconspicuous; vittae 3 in each furrow, ca. 4 on commissure. Seed face flat-concave (Figs. 4 and 5).

**Etymology.** The species is named after the spatulate petals of this plant.

**Distribution and habitat.***Tongoloa spathulata* is currently found in Yushu, located on the eastern Qinghai-Tibet Plateau. It grows on slope, at an altitude of 3600–3900 m. Fl. and Fr. Aug.– Oct.

#### *Tongoloa xinlongensis* L.J. Gui & X.J. He, sp. nov

**Type. CHINA. Sichuan**: Xinlong County. North of Pica Township, in shrubs and herbs near the damp place, 3573 m, 30°49′ N, 100°3′ E, 12 July 2020, Ling-jian Gui, GLJ20071201 (holotype: SZ!).

**Diagnosis.***Tongoloa xinlongensis* sp. nov. is similar to *T. spathulata*. However, this new species can be distinguished from the latter by its petals broad obovate, 2–2.2 × 1–1.2 mm (vs. spatulate, 1–1.5 × 0.4–0.8 mm), and the fruits are obviously larger (2.6–3 × 2.3–3 mm vs. 2.2 × 1.8).

**Fig. 6:**
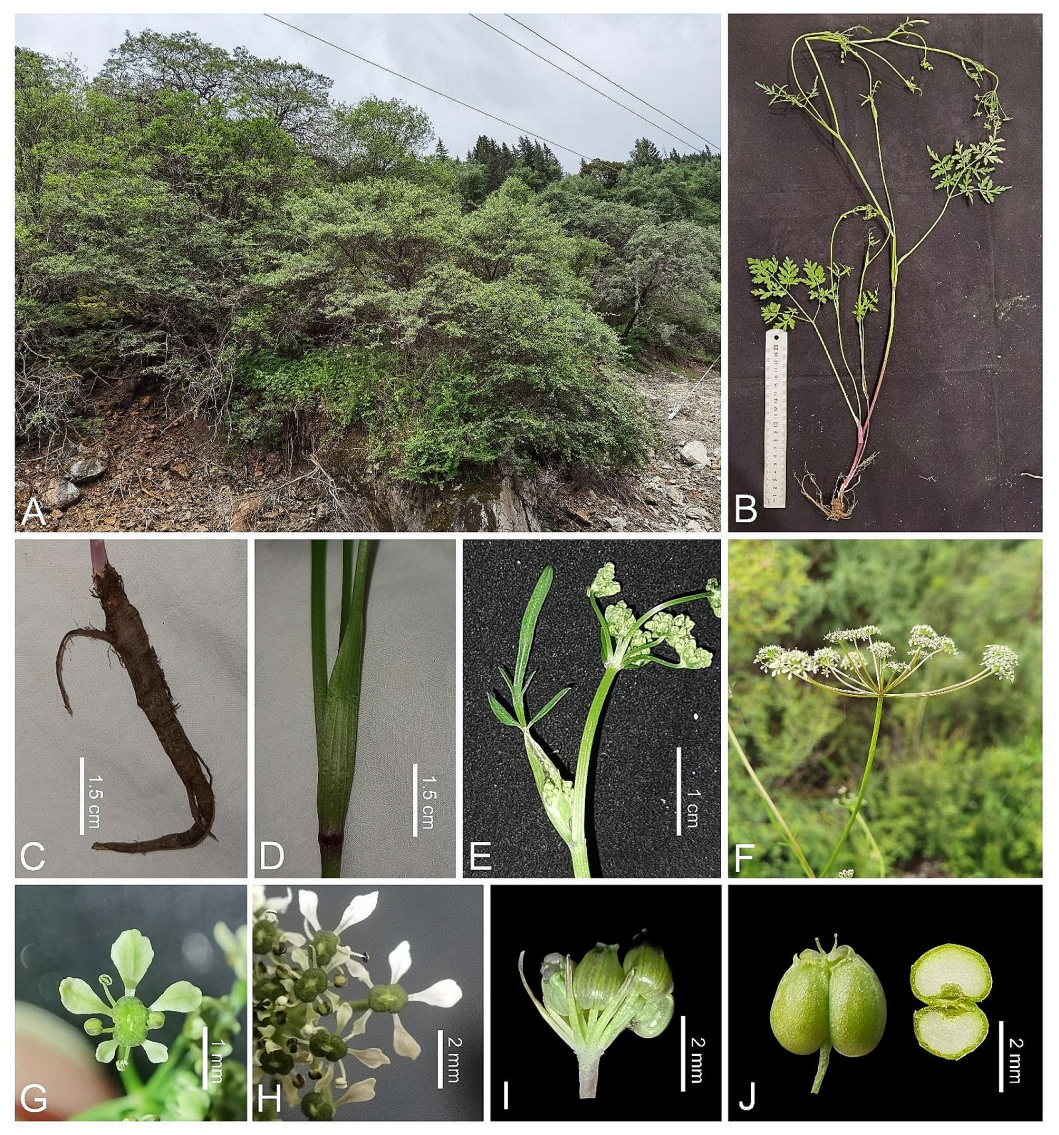
*Tongoloa xinlongensis* sp. nov. (**A**) Habitat; (**B**) Plant; (**C**) Root; (**D**–**E**) Membranous sheaths at the base of petioles; (**F**) Umbel; (**G**–**H**) Flowers; (**I**) Bracteoles; (**J**) Lateral and cross section view of mericarpes

**Fig. 7:**
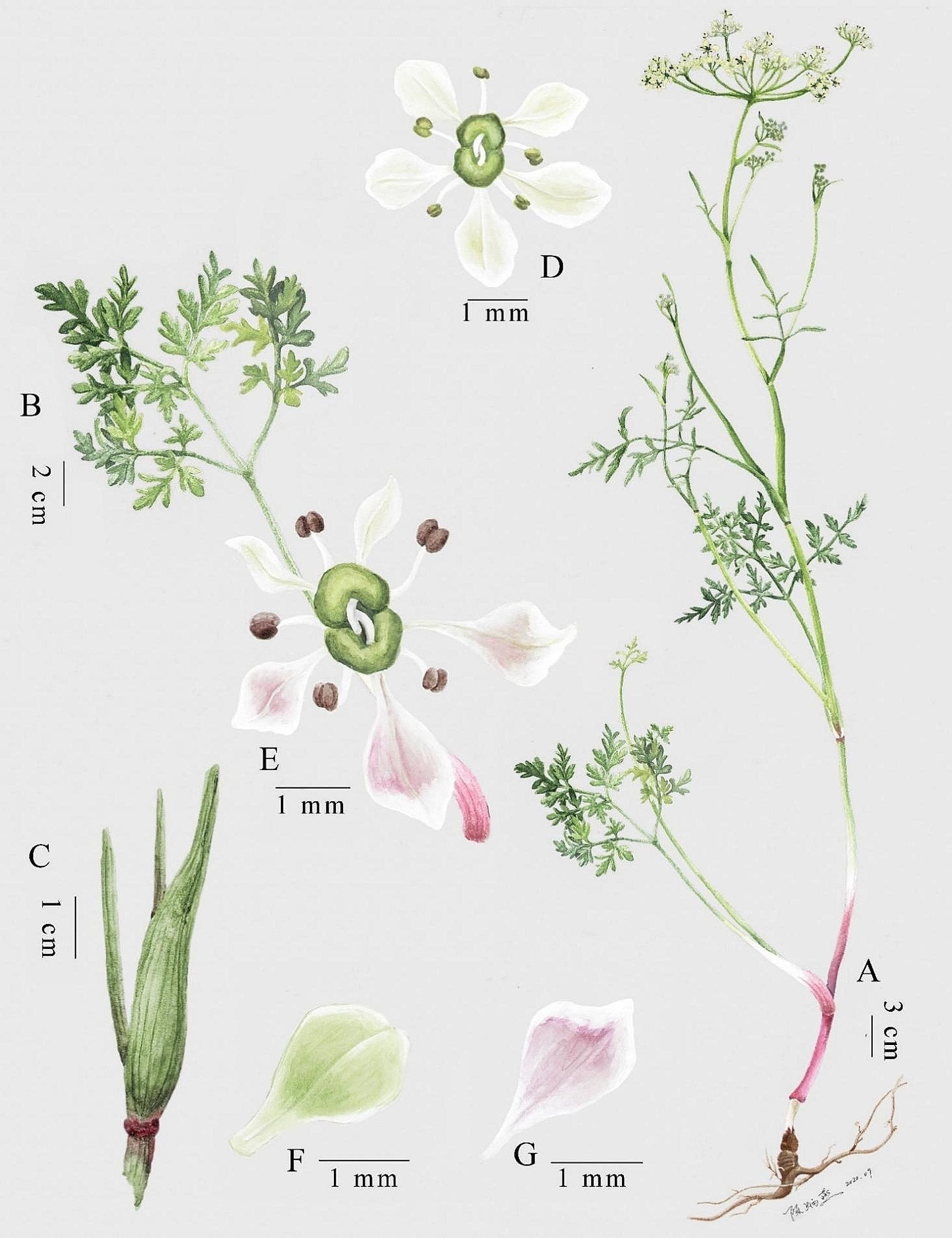
Scientific Illustration of *Tongoloa xinlongensis* sp. nov. (**A**) Plant; (**B**) Basal leaf; (**C**) Membranous sheath of petiole; (**D**–**G**) Flower. The apex of the petals is usually obtuse, but is pointed in a few individuals. Drawn by Bing-Yan Chen. Authorized for written publication

**Description.** Plants glabrous, 30–115 cm. Root conic. Stems ribbed and little branched, lower part purplish. Leaf sheaths membranous; blade triangular in outline, 5–14 × 4–10 cm, 2–3-ternate/pinnate, ultimate segments lanceolate-ovate, 3–15 × 2.5–4.5 mm, apex rounded or acute, margin sometiomes pinnate slightly; up petioles sheath-shaped, green. Umbels 3–15 cm across; bracts absent; rays 13–18; bracteoles usually absent or 1–3, linear, slightly longer than pedicels; pedicels unequal; umbellules 15–25-flowered. Calyx teeth minute and acute. petals obovate, apex usually obtuse-rounded, or with tips, white to purplish, ca. 2–2.2 × 1–1.2 mm. Anthers purple to greenlish. Stylopodium depressed, usually slanted inward in fruit period, greenlish to lavender; styles very short, reflexed. Fruit ovoid, base cordate, 2.6–3 × 2.3–3 mm, ribs filiform to inconspicuous; vittae 3 in each furrow, 4 on commissure. Seed face flat-concave (Figs. 6 and 7).

**Etymology.** The species epithet “xinlongensis” was named after Xinlong County, where the type materials were found.

**Phenology.** The species was observed flowering from July to September and fruiting from September to October.

**Distribution and habitat.***Tongoloa xinlongensis* grows in shrubs and herbs from 3200 m to 3800 m.

**Conservation status.***Tongoloa xinlongensis* is currently known to only occur in its type locality Xinlong County, Sichuan Province with several populations. We categorise it as Data Defcient (DD) according to IUCN (2019).

## Conclusion

*Tongoloa* is a genus within the East Asia Clade of Apiaceae, and the phylogeny reconstructed based on ITS sequences divides it into 3 main groups. These groups have similar fruit morphology. By integrating fruit morphology and molecular phylogenetic analyses, we preliminary clarified the intricate taxonomic relationships among *Tongoloa*, *Sinocarum*, Chinese *Pimpinella* subclade, *Sinolimprichtia* subclade, and Chinese *Trachydium* subclade. Furthermore, we proposed 2 new species, thereby suggesting a potentially richer species diversity of Apiaceae in the Hengduan Mountains.

### Electronic supplementary material

Below is the link to the electronic supplementary material.

Supplementary Material 1

## Data Availability

The ITS sequences can be downloaded from NCBI (https://www.ncbi.nlm.nih.gov) with accession numbers: OP422507-OP422516, MN630613-MN630619, MT124598-MT124620 and MZ054145-MZ054154.
