# Supplementary material for: Morphology and ITS sequences provide insights into the phylogeny of Tongoloa (Apiaceae) from China
Source: BMC Ecol Evol. 2024 Jul 30;24:103. doi: 10.1186/s12862-024-02292-5 (PMC11290071; doi:10.1186/s12862-024-02292-5)
Supplement: Supplementary file 1 — Supplementary Material 1 [file 12862_2024_2292_MOESM1_ESM.docx]

**Supplementary**

**Information**


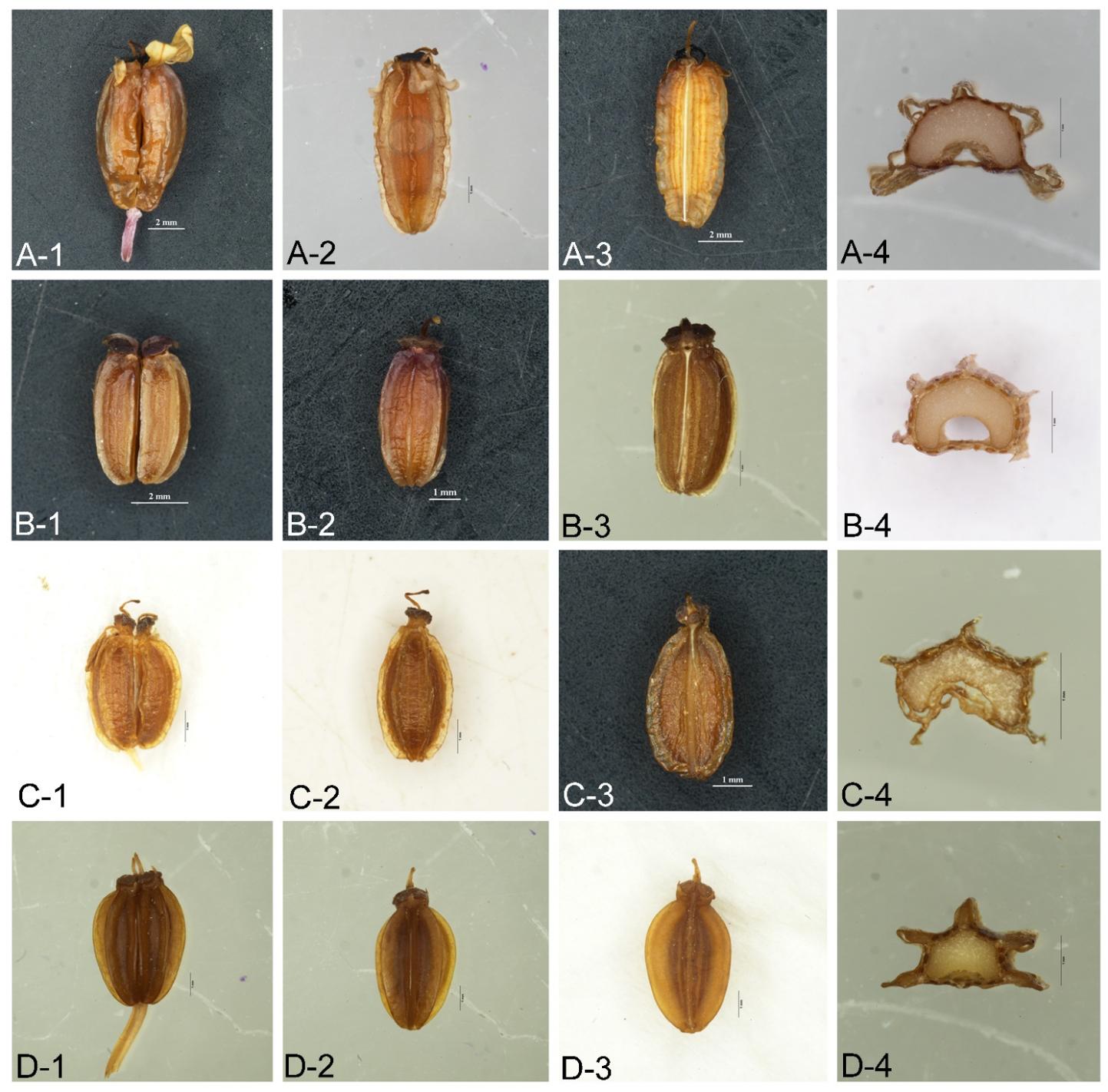


**Figure S1.** Fruit morphology of related genera. A. *Sinolimprichtia alpina* (GLJ19100702); B. *Pleurospermum amabile* (GLJ19100605); C. *Hymenidium lhasanum* (GLJ19092401); D. *Trachydium souliei* (GLJ19100604); 1. Lateral view; 2. Dorsal view; 3. Commissural view; 4. Cross section view. Voucher specimen enclosed in parentheses.


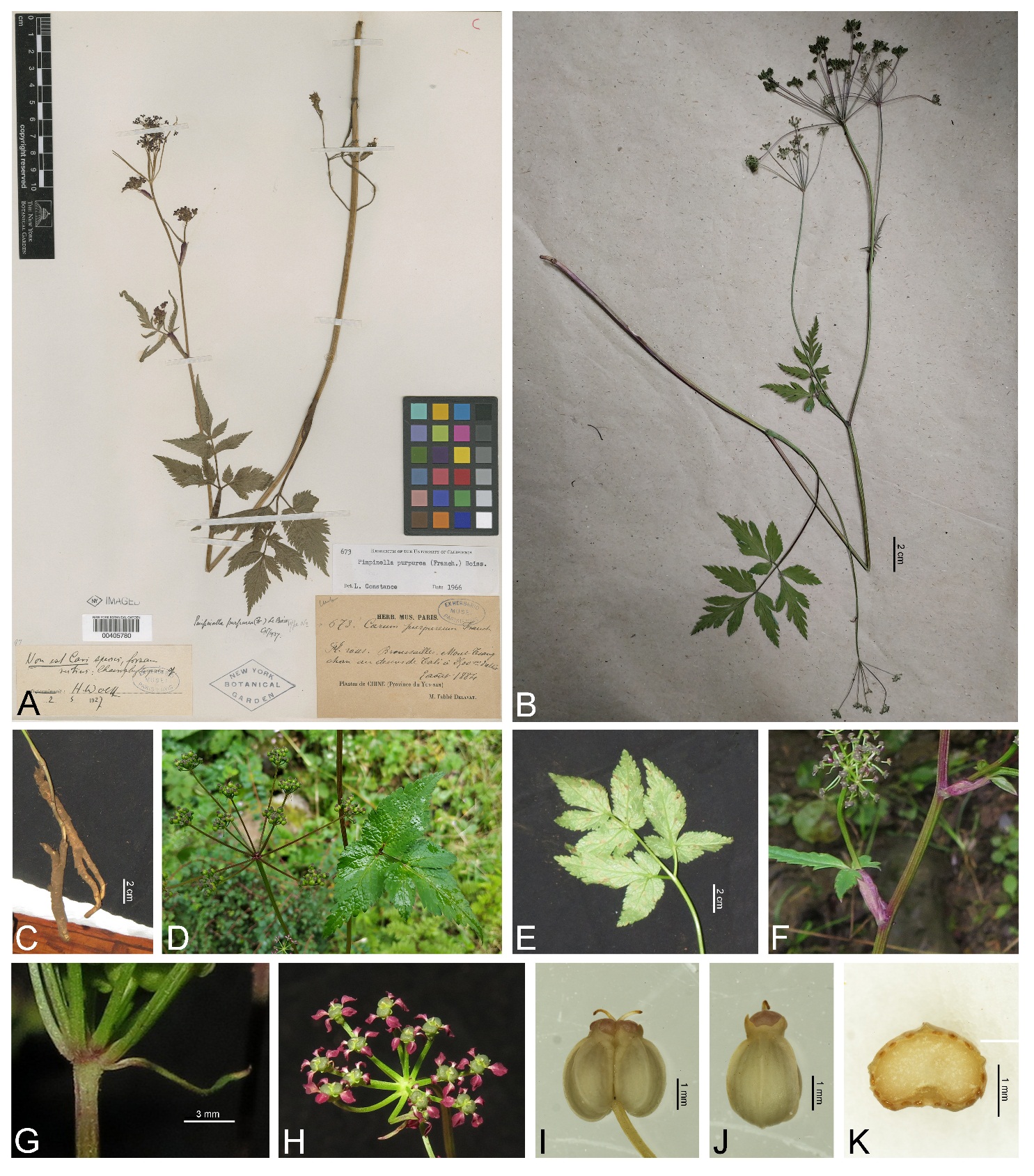


**Figure S2.** Morphology of *Pimpinella purpurea*. A. Isolectotype specimen (Delavay 673, barcode NY00405780); B. Specimen collected from Yunnan; C. Roots; D. plant; E. Lower leaf, dorsal view; F. Petiole sheath of upper leaves; G. Bracteole; H. Flowers; I. Fruit, lateral view; J-K. Mericarp, abaxial and cross section view.


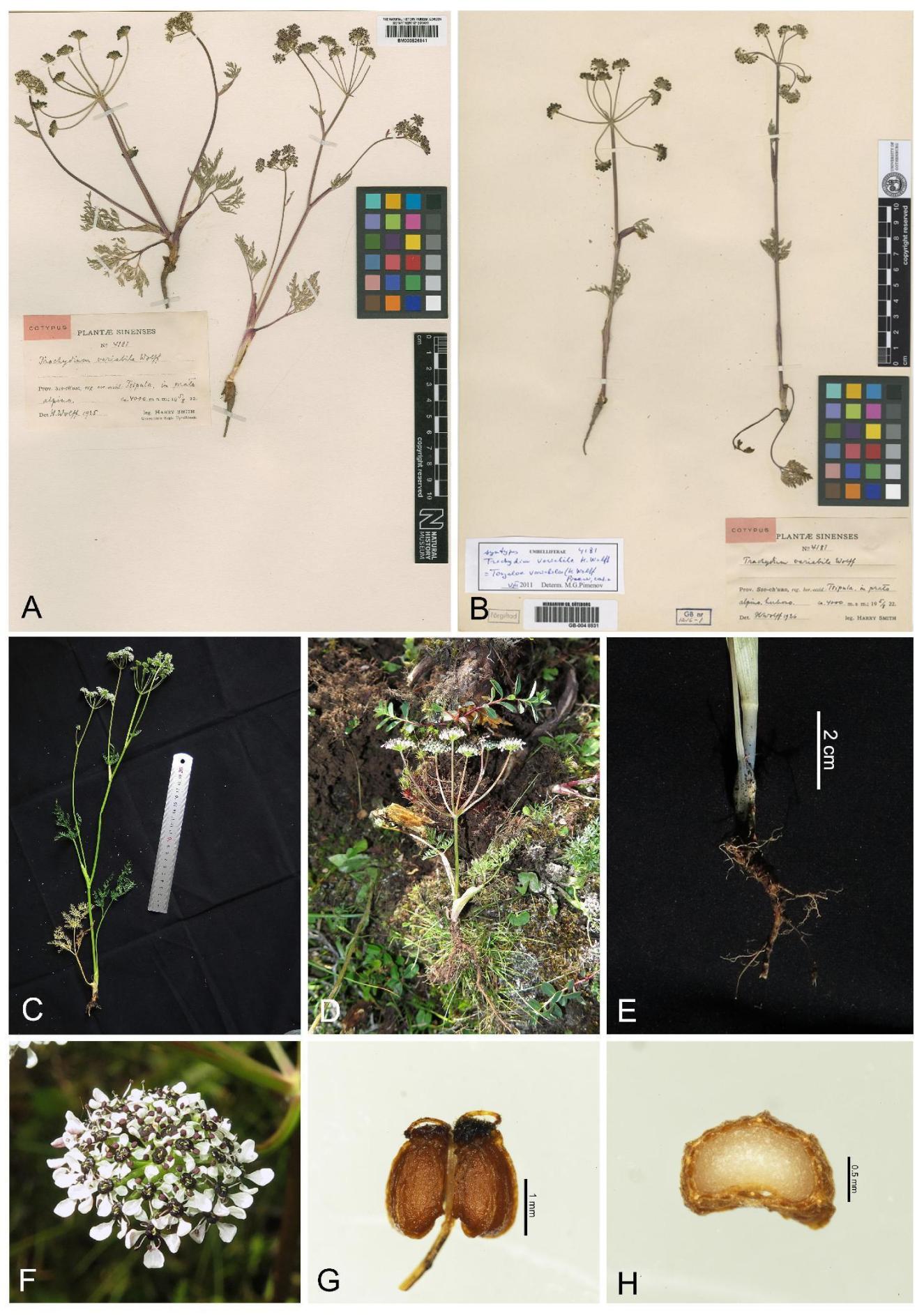


**Figure S3.** Morphology of *Trachydium variabile*. A. Syntype specimen (Harry Smith 4181, barcode BM000826941); B. Syntype specimen (Harry Smith 4181, barcode GB-0048831); C&D. Two types of plant morphology in different populations of this species; E. Short and woody root; F. Flowers; G. Lateral view of fruit; H. Cross section view of mericarp.

**
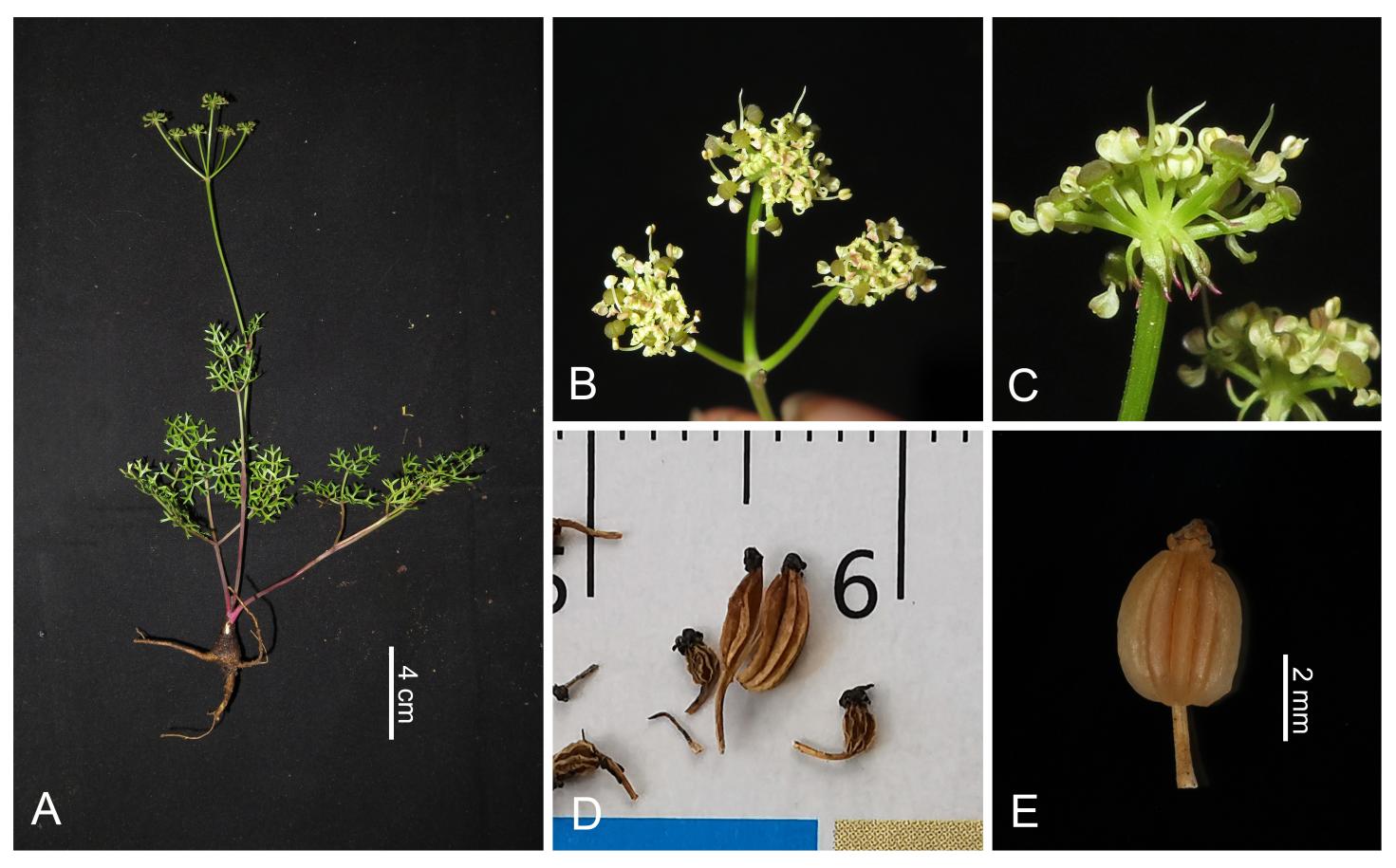
**

**Figure S4.** Morphological characteristics of samples collected from the type locality of *Tongoloa zhongdianensis*. A. Plant; B. Umbel; C. Flowers and bracteoles; D. Morphological comparison of mature fruits (large) and immature fruits (small) from the same individual; E. Dorsal view of mericarp.**Table S1.** The ITS sequences download from NCBI.

| ***Acromema* Clade** | *Sinocarum cruciatum*, MN846686; *Sinocarum coloratum*, MN846685; *Sinocarum muliensis*, MZ054145; *Acronema muscicola*, KP940756; *Acronema paniculatum*, KP940758; *Acronema astrantiifolium*, KP940757; *Sinocarum bellum*, MK309872; *Sinocarum ventricosum*, AY038200 & AY038214; *Oreocomopsis stelliphora*, GQ379322; *Ligusticum holopetalum*, MK088008; *Sinocarum wolffianum*, MK309871; *Meeboldia achilleifolia*, AY038206 & AY038220; *Meeboldia yunnanensis*, MN688999; *Meeboldia delavayi*, MN688992 |
| --- | --- |
| ***Hansenia* subclade** | *Haplosphaera phaea*, MT337432; *Hansenia forrestii*, MT337431; *Hansenia mongholica*, AF008643 & AF009122; *Hansenia weberbaueriana*, MN049521; *Haplosphaera himalayensis*, MT337433; *Hansenia oviformis*, MT337430; *Hansenia forbesii*, MN049518 |
| ***Hymenolaena* subclade** | *Hymenolaena* *badachschanica*, GQ379332; *Hymenidium nanum*, GQ379333; *Hymenolaena* *pimpinellifolia*, FJ469959 & FJ483498; *Pleurospermum amabile*, FJ469934; *Hymenolaena candollei*, FJ469958 & FJ483497; *Hymenidium lhasanum*, FJ469948 & FJ483487; *Pleurospermum amabile*, MT124614; *Hymenidium* *lhasanum*, MT124611; *Hymenidium amabile*, KP311487 |
| ***Komarovia* Clade** | *Parasilaus afghanicus*, MK088003; *Cyclorhiza peucedanifolia*, FJ385042; *Tetrataenium nepalense*, HQ686493; *Chuanminshen violaceum*, HQ185256; *Changium smyrnioides*, HQ185252; *Pterocyclus rotundatus*, MK078059; *Komarovia anisosperma*, AF077897; *Changium smyrnioides*, HQ185237 |
